# miR-21 modulates paclitaxel sensitivity and hypoxia-inducible factor-1α expression in human ovarian cancer cells

**DOI:** 10.3892/ol.2013.1432

**Published:** 2013-06-28

**Authors:** ZHONGBIN XIE, LIPING CAO, JUN ZHANG

**Affiliations:** 1 Department of Clinical Laboratory Medicine, Yulin Number Two Hospital, Yulin 719000, P.R. China; 2 Department of Obstetrics and Gynecology, Yulin Traditional Chinese Medical Hospital, Yulin 719000, P.R. China; 3 Department of Clinical Laboratory Medicine, 3201 Hospital, Hanzhong, Shanxi 723000, P.R. China

**Keywords:** ovarian cancer, miR-21, paclitaxel resistance, hypoxia-inducible factor-1α

## Abstract

Drug resistance is a major problem encountered in the treatment of ovarian cancer. Previous studies have demonstrated that in several types of cancer the overexpression of the multidrug resistance 1 (MDR1) gene is mainly associated with drug resistance. The present study aimed to investigate the role of miR-21 in the development of drug resistance in ovarian cancer cells. The expression levels of miR-21 in the ovarian cancer A2780 and A2780/taxol cell lines were detected by stem-loop real-time PCR. A2780 and A2780/taxol cells were transfected with mimics or inhibitors of miR-21 or negative control RNA. The expression levels of P-glycoprotein (P-gp) and hypoxia-inducible factor-1α (HIF-1α) proteins were assessed by western blot analysis. Drug sensitivity was analyzed by the 3-(4,5-dimethylthiazol-2-yl)-2,5-diphenyltetrazolium bromide (MTT) assay. The expression levels of miR-21 and P-gp were upregulated to a greater extent in the paclitaxel-resistant ovarian cancer A2780/taxol cell line compared with the parental A2780 cell line. Transfection of A2780/taxol cells with inhibitors of miR-21 decreased the expression levels of the P-gp and HIF-1α proteins, and increased the sensitivity of the A2780/taxol cells to paclitaxel. The expression levels of P-gp were additionally increased; however, the sensitivity of the miR-21 mimic-treated A2780 cells to paclitaxel was decreased. miR-21 may be involved in the development of drug resistance and the regulation of MDR1/P-gp expression, at least in part, by targeting HIF-1α in ovarian cancer cells.

## Introduction

Ovarian cancer is a common gynecological malignancy that occurs in females worldwide. Annually, >230,000 new cases of ovarian cancer are reported, causing >140,000 mortalities (1). Chemotherapy is the most effective primary therapy for the treatment of ovarian carcinoma, with initial response rates varying between 40 and 80% (2). However, numerous patients with ovarian cancer who initially respond to chemotherapy eventually relapse with a drug-resistant form of the disease (3). Thus, acquired resistance represents the major limitation to successful treatment. The molecular genetic basis of resistance to cancer treatment is complex and involves multiple processes, including drug transport and metabolism, DNA repair and apoptosis (4). Currently, the factors that regulate the development of chemoresistance in ovarian cancer remain poorly understood.

Paclitaxel is commonly used in the treatment of several types of cancer, including ovarian, breast and non-small cell lung cancer. It has also been used in pediatric patients with refractory malignancies and has been proposed as a potential agent against high-risk hepatoblastoma (5–7). Paclitaxel primarily kills cancer cells via microtubule stabilization; however, other mechanisms have been reported to mediate paclitaxel-induced cell death. It has been demonstrated that paclitaxel is able to induce mitochondrion stress through the activation of p38 (8). Several major mechanisms have been demonstrated to be important in the development of drug resistance to chemotherapy, including increased levels of repair to DNA damage, reduced apoptosis, altered drug metabolism and the overexpression of ATP-binding cassette (ABC) transporters (9,10). P-glycoprotein (P-gp) belongs to the ABC transporter family and its overexpression is considered to contribute to the development of drug resistance in numerous types of tumors, including ovarian cancer (11,12); however, the mechanism by which P-gp is overexpressed has yet to be elucidated.

The present study examined the role of miR-21 in the development of drug resistance in human ovarian cancer cells. The results demonstrated that aberrant miR-21 expression may be involved in the modulation of hypoxia-inducible factor-1α (HIF-1α) expression and the resistance of A2780 cells to paclitaxel.

## Materials and methods

### Cell lines and culture

Human ovarian cancer A2780 cell lines were purchased from the China Center for Type Culture Collection (Shaghai, China). The cell lines were cultured in RPMI-1640 medium (Gibco-BRL, Grand Island, NY, USA) supplemented with 10% FBS (Gibco-BRL, Melbourne, Australia) and 1% penicillin-streptomycin (Invitrogen Life Technologies, Carlsbad, CA, USA) and maintained at 37ºC in a humidified atmosphere of 5% CO_2_. The cells were passaged every 2–3 days. The establishment of paclitaxel-resistant ovarian cancer (A2780/taxol) cell lines was performed as described previously (13).

### miRNA transfection

The mimics and inhibitors of miR-21 were chemically synthesized by GenePharma Co., Ltd. (Shanghai, China). A2780 and A2780/taxol cells were seeded in 6-well plates at 3×10^5^ cells/well and cultured for 18 h. The cells were then transfected with 100 pmol of the miR-21 mimics, inhibitors or negative control (NC) RNA using Lipofectamine 2000 and Opti-MEM I reduced serum medium (Invitrogen Life Technologies), according to the manufacturer’s instructions.

### Stem-loop RT-PCR for the detection of miR-21 expression levels

To validate the differential expression levels of miR-21 in the A2780 and A2780/taxol cells, real-time RT-PCR analysis was performed. Stem-loop primers were used for the reverse transcription of miRNAs as described previously (14). The complementary DNA (cDNA) underwent 35 rounds of amplification (Bio-Rad S1000; Bio-Rad, Hercules, CA, USA) as follows: 35 cycles of a 2-step PCR (95ºC for 15 sec and 60ºC for 30 sec) following an initial denaturation (95ºC for 10 min) with 2 μl cDNA solution and 1X SYBR-Green Premix PCR reaction buffer (Takara Bio, Inc., Dalian, China). The sequence of primers used for the amplification was as described previously (14). Levels of miRNA were normalized using U6 RNA as an internal reference gene and compared with parent cells. The relative amount of miRNA to U6 RNA was examined using the 2^−ΔΔCt^ method (15).

### Cell viability assay

The cells were seeded into 96-well culture plates at a 5×10^3^ cell density. Following cellular adhesion, the A2780/taxol cells were exposed to 0.04, 0.2, 1.0, 5 and 25 μM doses of paclitaxel and the A2780 cells were exposed to 0.01, 0.05, 0.25, 1.25 and 6.25 μM doses of paclitaxel for 48 h. Following incubation, 20 μl of 5 mg/ml 3-(4,5-dimethylthiazol-2-yl)-2,5-diphenyltetrazolium bromide (MTT; Sigma, St. Louis, MO, USA) was added to each well. Following further incubation for 4 h at 37ºC, the medium was gently aspirated and replaced by 150 μl DMSO. The absorbance of each well was detected at a wavelength of 570 nm using a microplate reader (Bio-Rad). The experiments were conducted in triplicate.

### Small interfering RNA (siRNA) transfection

HIF-1α siRNA and a non-targeting control were purchased from Ambion (Applied Biosystems, Foster City, CA, USA) and the transfection was performed according to the manufacturer’s instructions. The cells were prepared for further analysis 48 h after the transfection. The transfection efficiency was evaluated by flow cytometry by calculating the percentage of fluorescein-labeled cells. The transfection efficiency was ~75%.

### Immunoblot analysis

The cells were harvested and washed with ice-cold phosphate-buffered saline. Cell lysates were obtained by re-suspending the cells in RIPA buffer [10 mM Tris (pH 7.4), 150 mM NaCl, 1% Triton X-100, 1% Na-deoxycholate (Kanto Chemical, Tokyo, Japan)] and 5 mM EDTA supplemented with protease inhibitor cocktail (Sigma). The protein concentration of the cell lysates was determined by BSA assay using the BSA kit (Beyotime, Shanghai, China). Equal amounts of protein were separated by SDS-PAGE and electrotransferred onto a PVDF membrane (Millipore, Billerica, MA, USA). The membranes were blocked and incubated overnight with P-gp, HIF-1α or GAPDH antibodies (Santa Cruz Biotechnology Inc., Santa Cruz, CA, USA), according to the manufacturer’s instructions. Signals present on the membrane were developed using the ECL reagent (Amersham, San Francisco, CA, USA) and were imaged using a polaroid imaging system (Amersham).

### Statistical analysis

Each experiment was repeated at least three times. Numerical data are expressed as the mean ± SD. Statistical analyses were performed using SPSS 12.0 software (SPSS, Inc., Chicago, IL, USA). P<0.05 was considered to indicate a statistically significant difference.

## Results

### Expression levels of P-gp and miR-21 in A2780 and A2780/taxol cells

**Figure 1:**
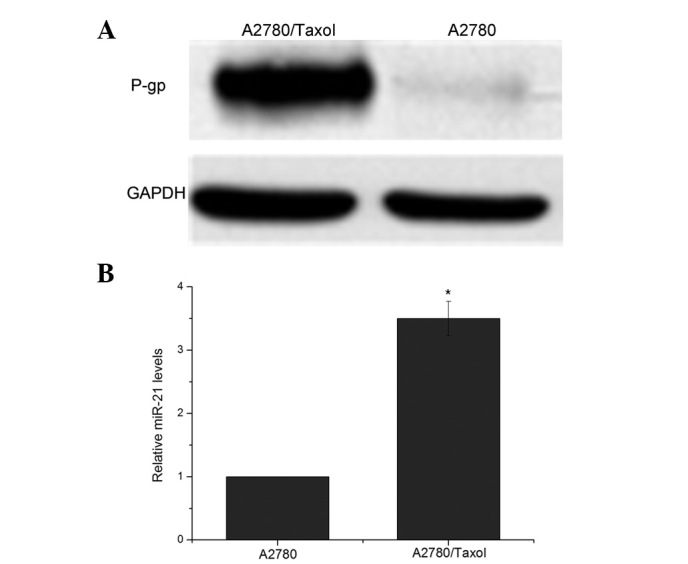
Expression levels of P-gp and miR-21 in A2780 and A2780/taxol cells. (A) P-gp protein levels in A2780 and A2780/taxol cells. (B) miR-21 levels in A2780 and A2780/taxol cells. ^*^P<0.05 vs. A2780 cells. P-gp, P-glycoprotein.

The overexpression of P-gp has been shown to contribute to the development of drug resistance in numerous types of cancer cell (16). In the present study, flow cytometry was used to measure the expression levels of P-gp in the A2780 and A2780/taxol cells. The expression levels of P-gp were increased in the A2780/taxol cell line compared with the parental A2780 cell line (Fig. 1A). Bourguignon *et al* demonstrated that high levels of P-gp were associated with high levels of miR-21 in drug-resistant breast cancer cells (17). In the present study, the expression levels of miR-21 in the A2780 and A2780/taxol cell lines were then detected using stem-loop real-time PCR. It was shown that the expression levels of miR-21 were on average 3.1-fold higher in the A2780/taxol cells compared with the A2780 cells (P<0.05; Fig. 1B).

### miR-21 modulates sensitivity to paclitaxel

**Figure 2:**
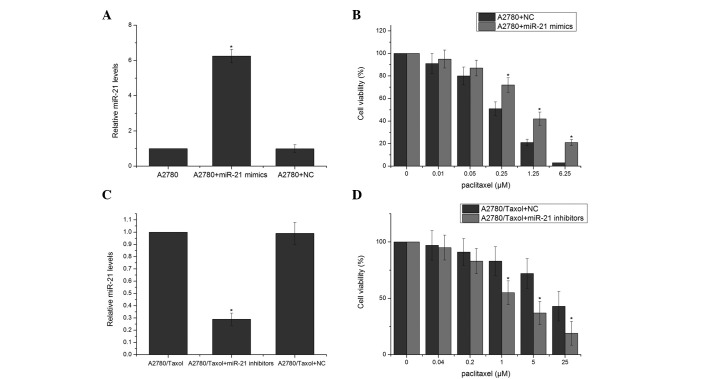
miR-21 modulates sensitivity to paclitaxel. (A) miR-21 levels in A2780 cells transfected with miR-21 mimics. (B) Paclitaxel sensitivity of A2780 cells transfected with miR-21 mimics or NC RNA. (C) miR-21 levels in A2780/taxol cells transfected with miR-21 inhibitors. (D) Paclitaxel sensitivity of A2780/taxol cells transfected with miR-21 inhibitors or NC RNA. ^*^P<0.05 vs. NC group. NC, negative control.

To further investigate whether miR-21 is capable of modulating the sensitivity of A2780/taxol and A2780 cells to paclitaxel, the A2780 and A2780/taxol cells were transfected with hsa-miR-21 and miR-21 inhibitors, respectively. In the A2780 cells, miR-21 mimics significantly increased the levels of miR-21 (Fig. 2A). The expression levels of miR-21 were decreased in the A2780/taxol cells transfected with miR-21 inhibitors (Fig. 2C). The MTT assay revealed that the cells transfected with miR-21 mimics exhibited a significantly increased resistance to paclitaxel compared with the negative control (NC) RNA-transfected cells (Fig. 2B). The A2780/taxol cells transfected with miR-21 inhibitors exhibited a significantly increased sensitivity to paclitaxel compared with cells transfected with NC RNA (Fig. 2D). These results suggested that miR-21 may modulate the sensitivity of A2780 cells to paclitaxel.

### Effect of miR-21 on the expression of multidrug resistance 1 (MDR1) and P-gp

**Figure 3:**
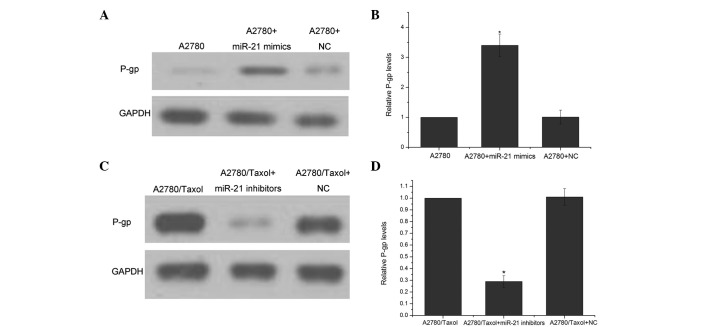
Effect of miR-21 on the expression levels of MDR1/P-gp. (A and B) P-gp protein levels in A2780 cells transfected with miR-21 mimics. (C and D) P-gp protein levels in A2780/taxol cells transfected with miR-21 inhibitors. ^*^P<0.05 vs. NC group. P-gp, P-glycoprotein; MDR1, multidrug resistance gene 1.

To determine whether miR-21 is capable of regulating the expression of MDR1/P-gp, the A2780 and A2780/taxol cells were transfected with mimics and inhibitors of miR-21, respectively, and the expression levels of P-gp were determined by western blot analysis. The transfection with miR-21 mimics resulted in increased expression levels of P-gp, whereas the transfection with NC RNA demonstrated no changes in the expression of MDR1/P-gp in the A2780 cells (Fig. 3A and B). To further test the effect of miR-21 on the expression of MDR1, the A2780/taxol cells were transfected with miR-21 inhibitors or NC RNA. The transfection with miR-21 inhibitors resulted in decreased levels of MDR1 mRNA (Fig. 2A) and P-gp expression (Fig. 3C and D).

### Regulation of HIF-1α expression by miR-21

**Figure 4:**
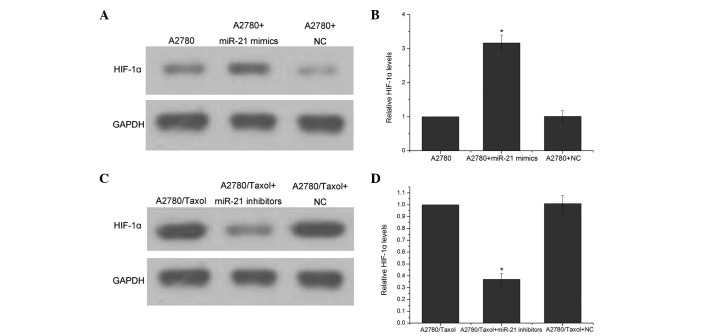
HIF-1α is regulated by miR-21. (A and B) HIF-1α protein levels in A2780 cells transfected with miR-21 mimics. (C and D) HIF-1α protein levels in A2780/taxol cells transfected with miR-21 inhibitors. ^*^P<0.05 vs. NC group. HIF-1α, hypoxia-inducible factor-1α.

HIF-1 is a heterodimeric transcription factor composed of two subunits: HIF-1α and HIF-1β. HIF-1α is induced by hypoxia, growth factors and oncogenes, whereas HIF-1β is constitutively expressed in cells (18). To test whether miR-21 affects HIF-1 expression, the A2780 and A2780/taxol cells were transfected with miR-21 mimics and inhibitors, respectively. It was demonstrated that the overexpression of miR-21 significantly increased the expression of HIF-1α in the A2780 cells (Fig. 4A and B). The treatment with miR-21 inhibitors decreased the expression levels of HIF-1α in the A2780/taxol cells (Fig. 4C and D).

### HIF-1α is important in paclitaxel resistance

**Figure 5:**
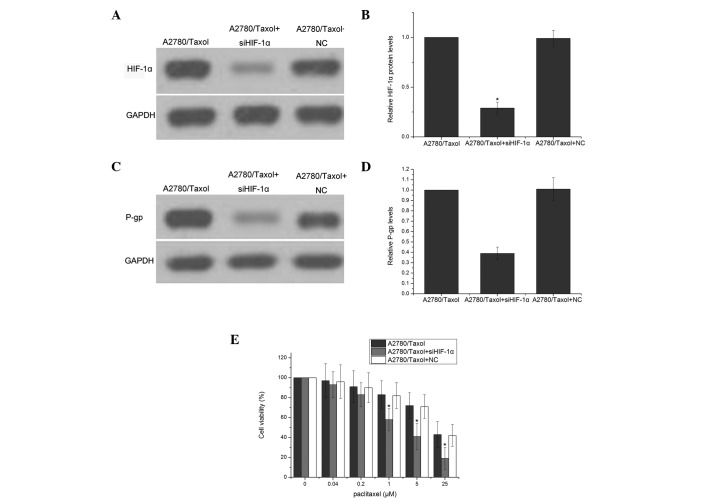
HIF-1α is important in paclitaxel resistance. (A and B) HIF-1α protein levels in A2780/taxol cells transfected with siHIF-1α or NC RNA. (C and D) P-gp protein levels in A2780/taxol cells transfected with siHIF-1α or NC RNA. (E) Paclitaxel sensitivity of A2780/taxol cells transfected with siHIF-1α or NC RNA. ^*^P<0.05 vs. NC group. HIF-1α, hypoxia-inducible factor-1α; NC, negative control; siHIF-1α, small interfering hypoxia-inducible factor-1α.

A previous study demonstrated that HIF-1α is involved in the development of drug resistance in several types of cancer (16), however, its role in paclitaxel sensitivity in the A2780 cell line remains unclear. To examine the correlation between HIF-1α and paclitaxel-induced cytotoxicity, HIF-1α siRNA or a scrambled siRNA was transfected into the A2780/taxol cells, followed by treatment with various doses of paclitaxel. HIF-1α siRNA significantly decreased the protein levels of HIF-1α (Fig. 5A and B). The protein levels of P-gp were decreased in the A2780/taxol cells transfected with HIF-1α siRNA (Fig. 5C and D). Furthermore, the A2780/taxol cells treated with HIF-1α siRNA exhibited a decreased survival rate compared with the control group (Fig. 5E).

## Discussion

Although chemotherapeutic agents, including paclitaxel, are widely used for the treatment of ovarian cancer, chemoresistance remains a major therapeutic obstacle (19). In the present study, cells from the human ovarian cancer A2780 cell line were used as targets to examine the effects of mRNA on reverse MDR of ovarian cancer cells in an attempt to identify novel treatment targets for ovarian cancer therapy. The results demonstrated that the knockdown of HIF-1α expression, in addition to decreased levels of miR-21, was capable of re-establishing the susceptibility of cancer cells to paclitaxel through the inhibition of P-gp expression, indicating that overexpression of miR-21/HIF-1α is important in the development of chemoresistance.

A recent study has indicated that miR-21 contributes to drug resistance in solid tumors and leukemia through several pathways. The inhibition of miR-21 may decrease cell growth, induce apoptosis and suppress migration and invasion in numerous types of cancer cell (20). Furthermore, several studies have demonstrated that the inhibition of miR-21 may sensitize leukemia cells to chemotherapy drugs (21,22). The present study demonstrated that miR-21 was upregulated to a greater extent in the A2780/taxol cells compared with the A2780 cells, indicating that miR-21 is involved in the development of paclitaxel resistance in ovarian cancer. Additional experiments involving the overexpression and underexpression of miR-21 were performed to confirm the effects of miR-21 on paclitaxel resistance in the A2780 cells. These demonstrated that the overexpression of miR-21 attenuated cell death, whereas the knockdown of miR-21 expression stimulated cell death.

HIF-1α is a multifunctional transcription factor, which has been shown to regulate tumor cell invasion and migration. Recent studies have demonstrated that HIF-1α contributes to the development of chemoresistance. Nardinocchi *et al*(23) demonstrated that HIPK2 is able to downregulate the expression of HIF-1α, which is overexpressed in several types of tumor and contributes to the development of chemoresistance by activating MDR1. In the present study, it was proposed that the HIPK2-mediated inhibition of HIF-1α correlated with the suppression of MDR1 gene transcription and the sensitization of cobalt-treated tumor cells to adriamycin-induced apoptosis. The results showed that HIF-1α and P-gp protein levels were significantly decreased in the A2780/taxol cells treated with inhibitors of miR-21 compared with the control group. The A2780/taxol cells that were pretreated with HIF-1α siRNA exhibited a decreased survival rate and decreased P-gp protein levels compared with the control group. Furthermore, the inhibition of miR-21 may sensitize A2780/taxol cells to paclitaxel-induced cell death. The miR-21/HIF-1α/MDR1/P-gp cell signaling pathway provides a novel insight into the underlying mechanisms responsible for paclitaxel resistance in A2780 cells.

In conclusion, the present study demonstrated that miR-21 may regulate the expression of MDR1/P-gp, at least in part, by targeting HIF-1α, which is involved in the development of drug resistance in paclitaxel-resistant ovarian cancer A2780/taxol cell lines. Furthermore, it was demonstrated that the inhibition of miR-21 may sensitize A2780/taxol cells to paclitaxel. This study may present a promising future strategy to reverse drug resistance through the targeting of miRNAs.
